# Long Non-Coding RNAs: Emerging and Versatile Regulators in Host–Virus Interactions

**DOI:** 10.3389/fimmu.2017.01663

**Published:** 2017-11-28

**Authors:** Xing-Yu Meng, Yuzi Luo, Muhammad Naveed Anwar, Yuan Sun, Yao Gao, Huawei Zhang, Muhammad Munir, Hua-Ji Qiu

**Affiliations:** ^1^State Key Laboratory of Veterinary Biotechnology, Harbin Veterinary Research Institute, Chinese Academy of Agricultural Sciences, Harbin, China; ^2^The Pirbright Institute, Woking, United Kingdom

**Keywords:** long non-coding RNAs, viral replication, antiviral response, virus–host interactions, regulatory mechanisms

## Abstract

Long non-coding RNAs (lncRNAs) are a class of non-protein-coding RNA molecules, which are involved in various biological processes, including chromatin modification, cell differentiation, pre-mRNA transcription and splicing, protein translation, etc. During the last decade, increasing evidence has suggested the involvement of lncRNAs in both immune and antiviral responses as positive or negative regulators. The immunity-associated lncRNAs modulate diverse and multilayered immune checkpoints, including activation or repression of innate immune signaling components, such as interleukin (IL)-8, IL-10, retinoic acid inducible gene I, toll-like receptors 1, 3, and 8, and interferon (IFN) regulatory factor 7, transcriptional regulation of various IFN-stimulated genes, and initiation of the cell apoptosis pathways. Additionally, some virus-encoded lncRNAs facilitate viral replication through individually or synergistically inhibiting the host antiviral responses or regulating multiple steps of the virus life cycle. Moreover, some viruses are reported to hijack host-encoded lncRNAs to establish persistent infections. Based on these amazing discoveries, lncRNAs are an emerging hotspot in host–virus interactions. In this review, we summarized the current findings of the host- or virus-encoded lncRNAs and the underlying mechanisms, discussed their impacts on immune responses and viral replication, and highlighted their critical roles in host–virus interactions.

## Introduction

**Figure 1:**
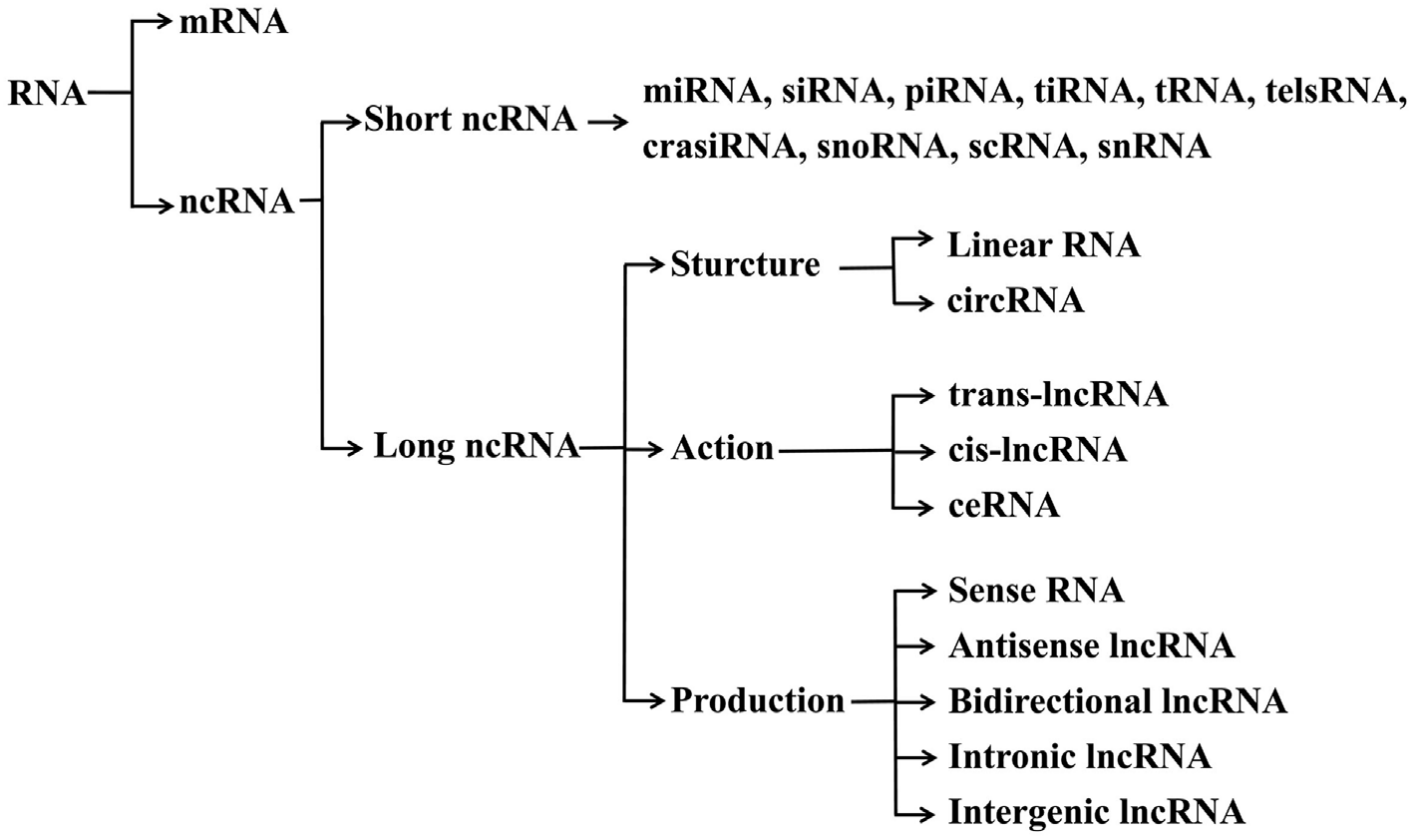
Classification of ncRNAs. mRNA, messenger RNA; ncRNA, non-coding RNA; miRNA, microRNA; siRNA, small interfering RNA; piRNA, piwi RNA; tiRNA, transcription initiation RNA; tRNA, transfer RNA; telsRNA, telomere-specific small RNA; crasiRNA, centromere repeat-associated small interacting RNA; snoRNA, small nucleolar RNA; scRNA, small cytoplasmic RNA; snRNA, small nuclear RNA; trans-lncRNA, *trans*-acting long non-coding RNA; cis-lncRNA, *cis*-acting long non-coding RNA; ceRNA, competing endogenous RNA.

With the rapid development of DNA sequencing technologies, the whole genomes of several species have been mapped and annotated. The first transcriptome analysis performed a decade ago came to a surprising conclusion that only about 2% of the genomic DNA harbors protein-coding genes (1). In the beginning of the 21st century, Okazaki et al. have analyzed the mouse transcriptome based on a cDNA library and identified a mass of non-coding RNAs (ncRNAs), which are defined as a class of RNA molecules without protein-coding capacity (2). In addition, the Encyclopedia of DNA Elements (ENCODE) project has widely been applied to identify the functional DNA elements in the human genome, and showed that approximately 62% of the transcriptome is ncRNAs (3, 4), indicating ncRNAs as major components of the transcriptome (5). In comparison with mRNAs, less is known about the functions and underlying mechanisms of ncRNAs in different biological processes. Based on the sequence length, ncRNAs are usually divided into long ncRNAs (lncRNAs, more than 200 nt) and short ncRNAs (sncRNAs, less than 200 nt) (6) (Figure 1).

In recent years, lncRNAs have been found to be critical regulators in various biological processes such as cell differentiation, chromatin modification, pre-mRNA transcription and splicing, and protein translation and translocation (7–9). Under a natural physiological state, lncRNAs usually function through enhancing or inhibiting the expression of neighboring protein-encoding genes (10). However, the investigation of potential roles of lncRNAs in virus–host interactions is still in the infancy stage. As a wide range of immunity-related lncRNAs has been identified based on differential expression analysis in response to viral infections, the host lncRNAs have been shown to act as regulators in the innate or adaptive immune signaling pathways (11, 12). Furthermore, emerging evidence demonstrates that viral genomes can transcribe their own lncRNAs by using the host transcription machinery, and these lncRNAs may be involved in the virus life cycle to regulate host or viral gene expression. Meanwhile, viruses can also regulate the expression of host lncRNAs to establish and maintain persistent infections.

For decades, studies on virus-related host immune responses have been focused mainly on genes or proteins. However, recent studies have shown that lncRNAs may also participate in these biological processes. This review will focus on the lncRNAs involved in host–virus interactions and underlying regulatory mechanisms.

## Sources and Functions of lncRNAs

Most of eukaryotic lncRNAs are transcribed by RNA polymerase II, whereas a limited number of lncRNAs are transcribed by cellular RNA polymerase III (13). After transcription and modification processes, some mature lncRNAs have a similar structure to that of mRNA, including methylguanosine at 5′-terminus and a polyadenylated [poly(A)] tail at the 3′-terminus (13, 14). Indeed, broader analysis has suggested that 39% of lncRNAs transcripts contain one or more of the six most common poly(A) motifs, compared with 51% observed for coding transcripts (13). These properties indicate that there are few particular structural features that allow differentiation of lncRNAs from mRNAs. Nevertheless, compared with mRNAs, lncRNAs are more specific in spatial expression and poorly conserved (15, 16). To date, five possible sources of lncRNAs have been verified: (1) DNA fragments can be assembled and transformed into a functional lncRNA; (2) due to chromosomal rearrangement, two or more mutually independent sequences link together to generate a lncRNA; (3) due to retrotransposition, duplication of non-coding genes can generate functional or non-functional lncRNAs; (4) duplication events from two neighboring tandems give rise to a sequence repeat lncRNA; (5) insertion of a transposable element in a gene generates a lncRNA (17).

**Figure 2:**
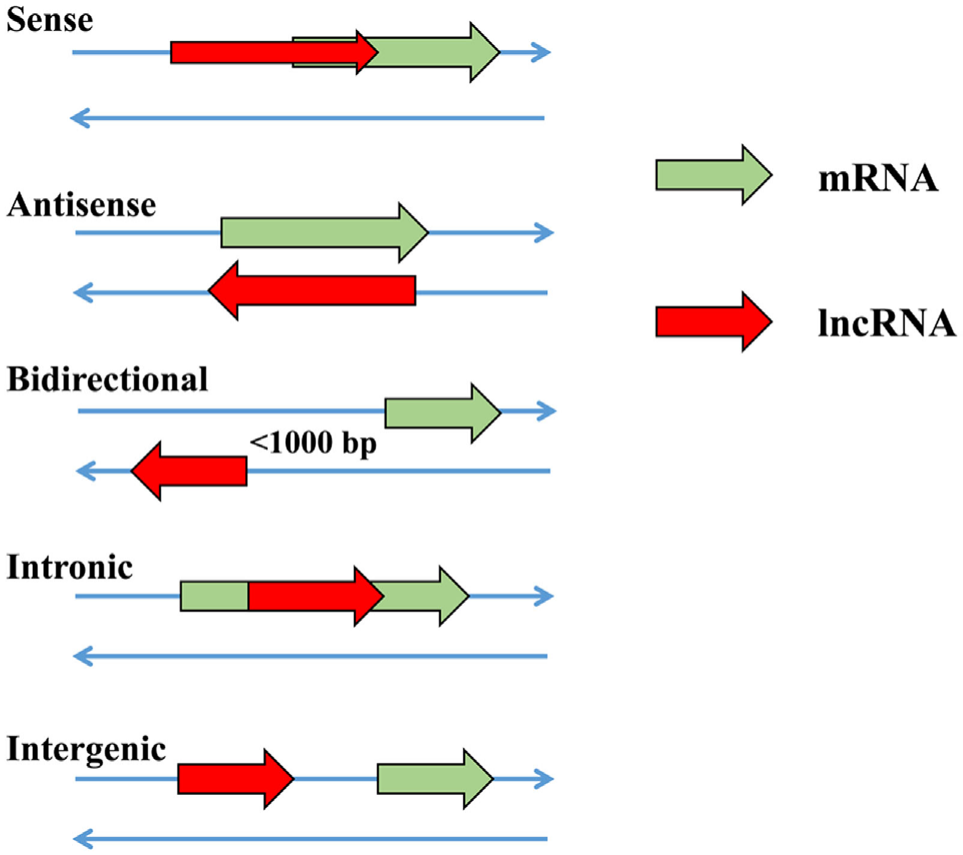
The relative positions of functional long non-coding RNAs (lncRNAs) and target protein-coding messenger RNAs (mRNAs).

In recent years, lncRNAs have been confirmed as a novel group of regulatory molecules in a wide range of biological or cellular processes (17–21). In the nucleus, lncRNAs participate in regulating the expression of nearby and overlapping genes in either RNA-independent or transcription-initiation manner after epigenetic modification (22). The lncRNA HOTAIR has been proved to repress gene expression by recruiting the histone protein (20). lncRNAs may function as enhancers to promote the expression of nearby genes (23–25). At the promoter regions, lncRNAs overlap with DNA sequence and assist the gene to maintain the transcriptional condition, which may be a common function *in cis*-regulation (26). In addition, lncRNAs can competitively bind to miRNAs to prevent the degradation or repression of target mRNA (27, 28). Based on transcriptional directions and relative positions with target mRNAs, lncRNAs are usually classified into five major categories, i.e., sense lncRNAs, antisense lncRNAs, bidirectional lncRNAs, intrinsic lncRNAs and intragenic lncRNAs (29) (Figure 2). The antisense lncRNAs comprise a significant proportion (almost 20%) of the total lncRNAs in mammalian genomes and 75% antisense lncRNAs are able to upregulate the expression of adjacent genes (30). In addition, more than 50% of protein-coding genes carry a complementary lncRNAs in mammals (31).

## Antiviral Activities of Host lncRNAs

### lncRNAs Are Involved in the Innate Immune Responses against Viral Infections

As mentioned above, diverse biological processes in eukaryotic cells are regulated by lncRNAs. However, it is noteworthy that viral infections may lead to the differential expression of host lncRNAs and this change seems to exist as a common pathological phenomenon (32–36). Some differentially expressed host lncRNAs may exert antiviral actions involved in different immune signaling pathways. Guttman et al. reported the modulation of lncRNAs transcription by regulatory proteins for the first time and uncovered over 100 lncRNAs with potential functions in four mouse cell types, i.e., mouse embryonic stem cells, mouse embryonic fibroblasts, mouse lung fibroblasts, and neural precursor cells, by using chromatin immunoprecipitation and massive parallel sequencing (37). Furthermore, it has also been confirmed that the transcription of lncRNAs is associated with immunity-related factors, such as nuclear factor κB (NF-κB) (39 lncRNAs), sex-determining region of Y chromosome-related high-mobility-group box 2 (Sox2) (20 lncRNAs), and p53 (118 lncRNAs). With the widespread applications of microarray and RNA sequencing technologies, differentially expressed lncRNAs have been identified to be involved in innate immune responses (32, 38–43).

#### lncRNAs Regulate the Interferon (IFN) Pathway of the Innate Immune Response

The lncRNA nuclear enriched abundant transcript 1 (NEAT1) is a well-defined positive regulatory component in interleukin (IL)-8 signaling pathway, which can activate the antiviral response. Influenza virus, human immunodeficiency virus (HIV), and other viral infections induce the expression of NEAT1, leading to the formation of nuclear body paraspeckles (44, 45). Splicing factor proline/glutamine-rich (SFPQ) is a negative regulatory factor of IL-8. NEAT1 mediates the relocation of SFPQ from the IL-8 promoter region to paraspeckles and activates the transcription of IL-8 (46). Although the exact antiviral mechanism of IL-8 is not clear, the concentration of IL-8 is proportional to the resistance against HIV infection in a macaque model (47). Moreover, NEAT1, as a binding scaffold, maintains integrity of paraspeckles and prevents the export of spliced pre-mRNA to the cytoplasm for translation. During HIV infection, the upregulated NEAT1 sequesters HIV mRNAs within the nucleus and inhibits viral replication (34). Another study shows that NEAT1 is significantly upregulated postinfection with Hantaan virus (HTNV), whereas inhibiting the expression of NEAT1 delays host innate immune responses and promotes viral replication (48). Further investigations indicate that NEAT1 removes and relocates SFPQ to paraspeckles, inducing the expressions of retinoic acid inducible gene I (RIG-I) and DEXDH box helicase (DDX60). Increased expression of DDX60 and RIG-I enhances IFN-β production and subsequently suppresses HTNV infection.

The lncRNA Cox2, located at 50 kb downstream of the Cox2 protein coding gene, regulates the activation and repression of hundreds of genes (36). It has been revealed that 787 genes are repressed by the lncRNA Cox2 in non-stimulated bone marrow-derived macrophages and 713 genes are expressed following exposure to toll-like receptor (TLR) 1/2 agonist palmitoy-3-cysteinyl-seryl-(lysyl)_4_ (Pam3CSK_4_) (41). The subsequent gene ontology (GO) analysis has revealed that the differentially expressed genes are involved in the regulation of immune responses. The whole transcriptome profiling has proven that Cox2 is in charge of activating and inducing interferon regulatory factor 7 (IRF7) and IL-10 and repressing TLR1, 3, and 8, which regulates the expression of various genes in both positive and negative regulatory manners (41). Although the exact regulatory mechanisms remain unknown, researchers speculated that the inhibitory actions of Cox2 could be mediated through binding to heterogeneous nuclear ribonucleoprotein (hnRNP)-A/B and hnRNP-A2/B1. Collectively, lncRNA Cox2 is a key regulatory factor of the circuit adjusting the TLR signaling pathway.

#### lncRNAs Mediate Other Pathways of the Innate Immune Response

Tumor necrosis factor-alpha (TNF-α) is a significant activator of host immune responses to viral infections (49–51). Recently, it has been shown that TNF-α is regulated by a lncRNA, TNF-α and hnRNPL- immunoregulatory lncRNA (THRIL) (38). The THRIL is located downstream of BRI3-binding protein (BRI3BP) and partially overlapped with the 3′-terminus of BRI3BP. This lncRNA THRIL is an essential factor for the induction of TNF-α gene expression by forming a complex with hnRNPL at the promoter/enhancer region of TNF, resulting in the activation of immune response genes (38). On the other hand, THRIL can also be downregulated by the activated TNF through a negative feedback mechanism. These findings highlight a wider spectrum of lncRNA roles in several cellular processes and warrant future investigations.

#### lncRNAs Participate in the Regulation of the Expression of Interferon-Stimulated Genes (ISGs)

ISGs are induced through the IFN signaling pathway and critical for antagonizing viral infections (52). To date, new antiviral ISGs are discovered as antiviral effectors in the innate antiviral responses (53, 54). In addition, ISGs have been confirmed to have numerous antiviral functions, such as interfering with and inhibiting viral infections, and limiting viral replication within the cells (52). However, molecular mechanisms of regulation of the ISGs expression are complicated (53, 55). Currently, several studies demonstrate that lncRNAs are the key regulators of ISGs.

Some viruses can induce the expression of the lncRNA BISPR (BST2 IFN-stimulated positive regulator) through the JAK-STAT pathway, such as influenza virus, vesicular stomatitis virus or hepatitis C virus (HCV) (56–59). BISPR is located head-to-head with the ISG BST2 gene, the BST2 protein can attach viruses to the cells and inhibit viral release (60, 61). Knockdown or overexpression of BISPR results in a decrease or increase of BST2 expression, respectively, suggesting that BISPR is critically responsible for the transcription of BST2. BISPR exists mainly in the nucleus and possibly facilitates the transcription initiation of protein-coding genes. As mentioned above, some lncRNAs regulate the chromatin state through recruiting and binding to various chromatin-modifying factors. Likewise, BISPR performs its regulatory function by counteracting the repressive action of polycomb repression complex 2 (PRC2) at the promoter of BST2, and the methyltransferase component of EZH2 is also involved in this mechanism (56). In addition, BISPR overlaps with an enhancer region, indicating that BISPR acts as enhancer-associated RNAs (eRNAs) to promote the formation of enhancer-promoter complex.

A functional lncRNA, called negative regulator of antiviral response (NRAV), is downregulated dramatically during influenza A virus (IAV) infection (62). Overexpression of NRAV in human cells or transgenic mice significantly increases IAV replication and virulence, whereas knockdown of NRAV suppresses IAV replication, indicating that NRAV is involved in antiviral immune responses. A cDNA microarray analysis reveals that many ISGs are downregulated in NRAV-overexpressing cells, such as IFIT2, IFIT3, IFITM3, OASL, and MxA, and these ISGs exert antiviral effects through multiple mechanisms (63–66). A subsequent study indicates that NRAV negatively regulates the initial transcription rates of IFITM3 and MxA through altering histone modifications (active H3K4me3 and repressive H3K27me3) on the promoters, and the spatial structure of NRAV is necessary for its regulatory function (62).

The lncRNA CMPK2 is located proximally to the ISGs CMPK2, which is mapped to chr2p25.2 (chr2:6,968,644-6,980,595). The lncRNA CMPK2 can be upregulated significantly by IFN-α or IFN-γ (25, 67). Knockdown of lncRNA CMPK2 in hepatocytes results in remarkable reduction in HCV replication and increases expression of some antiviral ISGs, suggesting that the lncRNA CMPK2 is a critical repressor of ISGs and a lncRNA-mediated negatively regulatory mechanism may exist. In addition, the level of the lncRNA CMPK2 is dramatically higher in the liver of HCV-infected patients compared with healthy donors, indicating that the lncRNA CMPK2 also plays a regulatory role in viral infections *in vivo* (25), whereas overexpression of the lncRNA CMPK2 inhibits the transcription of ISGs, such as CMPK2 and viperin. Interestingly, some ISGs located far from the lncRNA CMPK2 in the genome can also be repressed, such as ISG15, IFIT1, IFIT3, CXCL10, MxA, and IFITM1. Nevertheless, a few of ISGs seem to inhibit the transcription of the lncRNA CMPK2, including IFIT1 and Mx1. However, the impact of silencing of the lncRNA CMPK2 on ISG levels is not consistent with other IFN-stimulated negative regulatory factors, such as activating signal cointegrator 1 complex subunit 3. Thus, it is considered that the regulatory mechanism of lncRNA CMPK2 may be similar to other lncRNAs, such as NRAV. Similarly, lncRNA CMPK2 interacts with transcription factors or chromatin to form complexes to regulate the gene expression.

**Figure 3:**
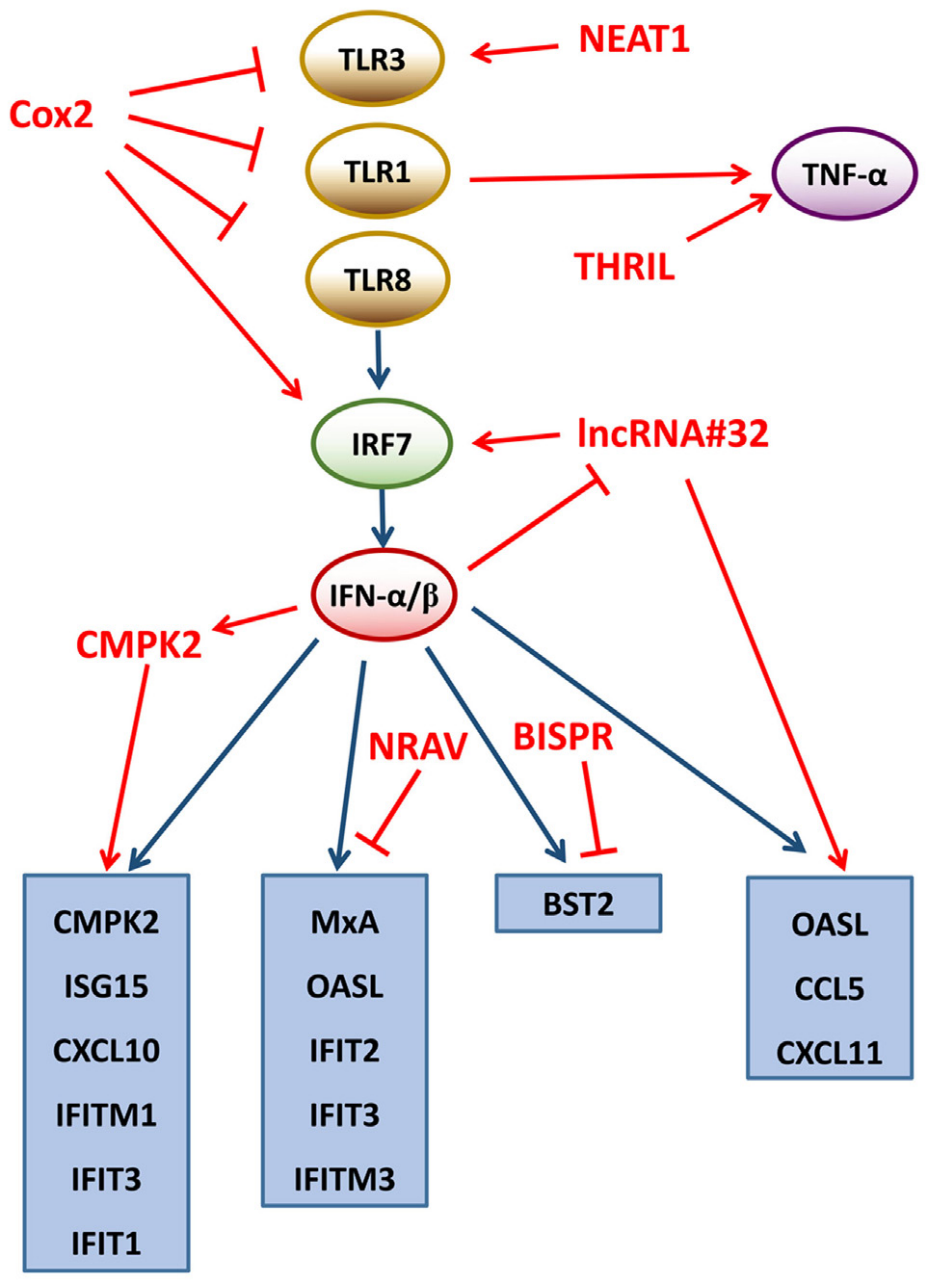
lncRNAs regulate the immune responses. Proteins and lncRNAs involved in the immune responses are shown in black and red, respectively. Inhibition is shown with a T-shaped line. Activation is depicted with an arrow. NEAT1, nuclear enriched abundant transcript 1; THRIL, TNF-α and hnRNPL-related immunoregulatory lncRNA; NRAV, negative regulator of antiviral response; BISPR, BST2 IFN-stimulated positive regulator; CMPK2, cytidine monophosphate kinase 2; TLR1, 3, and 8, toll-like receptors 1, 3, and 8; IRF7, interferon regulatory factor 7; TNF-α, tumor necrosis factor-alpha; IFN-α/β, interferon-alpha/beta; ISG15, interferon-stimulated gene 15; CXCL10 and 11, chemokine (C-X-C motif) ligand 10 and 11; IFIT1, 2, and 3, interferon-induced proteins with tetratricopeptide repeats 1, 2, and 3; IFITM 1 and 3, interferon-induced transmembrane protein 1 and 3; OASL, oligoadenylate synthetase-like; MxA, myxovirus resistance protein A; BST2, bone marrow stromal cell antigen 2; CCL5, chemokine (C-C motif) ligand 5; lncRNA, long non-coding RNA.

The lncRNA#32 is located on human chromosome 7p13 and overlaps the 3′-terminus of the HECT, C3, and WW domain containing E3 ubiquitin protein ligase 1 (HECW1) (68). Silencing lncRNA#32 significantly reduces the expression level of some ISGs and chemokines, including IRF7, chemokine (C-C motif) ligand 5 (CCL5), CXCL11, OASL, RSAD2, and IP-10, resulting in susceptibility to encephalomyocarditis virus (EMCV) infection. In contrast, the overexpression of lncRNA#32 dramatically suppressed EMCV replication, indicating that lncRNA#32 positively regulates the host antiviral response (68). The expression of OASL is induced by IFN-β, whereas the expression of lncRNA#32 is repressed by IFN-β in a dose-dependent manner. lncRNA#32 positively regulates the expression of ISGs through its interaction with activating transcription factor 2 (ATF2). The ATF2-binding region deletion mutant of lncRNA#32 does not induce IP-10 expression. The research also finds that heterogeneous nuclear ribonucleoprotein U (hnRNPU) maintains the expression of these ISGs by binding to and stabilizing lncRNA#32. These findings highlight the possibility that the hnRNPU-lncRNA#32 complex may target promoters of ISGs to promote the transcription (Figure 3).

Taken together, current understandings propose the nature and breadth of lncRNAs in the regulation of ISGs, which define the first line of defense against pathogens. While a significant baseline has been made, extensive future studies are required to underpin this important aspect of host-pathogen interactions along with their impacts on virus biology and host responses.

### lncRNAs Are Involved in the Adaptive Immune Response

Although the existence of lncRNAs in T cells has been known for years, such as growth-arrest-specific transcript 5 (Gas5) and non-coding transcript in CD4^+^ T cells, the lncRNA screening has recently been conducted in CD8^+^ T cells (69). A total of 1,524 lncRNAs were identified from 42 mouse T cell subsets using a microarray assay and some of them were lymphoid-specific lncRNAs, which were increased during CD8^+^ T cell activation and differentiation into effector T cells (70). At the differentiation state of CD4^+^ T cells to T_H_1 or T_H_2 subsets, T_H_1-related transcription factors, such as STAT4 and T-box transcription factor, can induce the expression of some T_H_1-specific lncRNAs. Likewise, T_H_2 transcription factor STAT6 regulates T_H_2-specific lncRNAs expression. In addition, lncRNA Gas5 represses T cell proliferation. Overexpression of Gas5 inhibits cell-cycle progression and initiates the cell apoptosis signaling pathways (71). Limited studies have been conducted to investigate the roles of lncRNAs in adaptive immune responses; however, current evidences propose crucial roles of lncRNAs in regulation of adaptive immunity and thus warrant future investigations.

## Host lncRNAs are Hijacked by Various Viruses

Host lncRNAs have been confirmed as positive or negative antiviral regulators in the immune response; surprisingly, a few of host lncRNAs can be induced and hijacked by certain viruses to establish persistent infections. This is likely due to the mutual adaptability of hosts and viruses for millions of years.

The lncRNA NeST, also known as Tmevpg1 or IfngAS1, is located adjacent to the IFN-γ gene in both humans and mice that can positively regulate the expression of IFN-γ (72). NeST can bind to WD repeat-containing protein 5 (WDR5), a component of histone H3 lysine 4 (H3K4) methyltransferase complex, and alter histone 3 methylation at the IFN-γ locus, resulting in the IFN-γ expression (72). In addition, the transcription of both mouse and human NeST gene is dependent on NF-κB and transcription factors STAT4 and T-bet (73, 74). An earlier study has shown that NeST is specially expressed in T_H_1 CD4^+^ T cells and is considered to be associated with immune response (73). Another similar study has indicated that NeST facilitates Theiler’s virus infection (75), which is verified using B10.S and SJL/L mouse models. The SJL/L mice with NeST gene show increased IFN-γ expression in activated CD8^+^ T cells, leading to persistent infection of Theiler’s virus, and the NeST gene-knockout B10.S mice can clear the virus by its own immune system. The transgenic B10.S mice carrying the allele of NeST are unable to resist the viral infection either. Thus, Theiler’s virus establishes persistent infections by hijacking the host lncRNA NeST.

The lncRNA NRON is required to regulate the activity of nuclear factor of activated T cells (NFAT) by forming a ribonucleoprotein complex with NFAT kinases and expression of this lncRNA is significantly altered following HIV-1 infection (45, 76–79). The regulation of NRON expression during the HIV-1 life cycle is complex. The level of NRON is reduced by the HIV-1 early accessory protein Nef and the dephosphorylated NFAT can be translocated to the nucleus and activates the expression of several genes of HIV (78). Knockdown of NRON enhances virus replication through increasing the activity of NFAT. However, high-level expression of NRON is induced by the HIV-1 accessory protein Vpu at the late stages of HIV infection, resulting in viral release and apoptosis. It has been demonstrated that the expression level of NRON is modulated by the HIV-1 Nef and Vpu proteins at different times postinfection to fit the virus life cycle. This finding explains how HIV regulates the host lncRNA NRON to facilitate viral infection.

lncRNA-ACOD1, located near the ACOD1 protein-coding gene, can be induced by various viruses, including Sendai virus (SeV), vesicular stomatitis virus (VSV), herpes simplex virus (HSV), and vaccinia virus (VACV) (80). In addition, lncRNA-ACOD1 is an IFN-α-independent lncRNA, of which expression is regardless of IFN-α receptor deficiency and IFN-α stimulation. Knockdown of lncRNA-ACOD1 significantly reduces viral load of VSV in macrophages and VSV replication is remarkably reduced in the lncRNA-ACOD1-deficient mice, indicating that the lncRNA promotes virus replication (80). Microarray transcriptome analysis shows that the lncRNA-ACOD1 deficiency leads to changes in the expressions of many metabolism-related genes, indicating the potential role of the lncRNA in regulation of metabolism upon viral infection. RNA immunoprecipitation assay suggests that lncRNA-ACOD1 directly binds to the metabolic enzyme glutamic-oxaloacetic transaminase 2 (GOT2) near the substrate niche, enhancing its catalytic activity. It has been shown that lncRNA-ACOD1 overexpression promotes viral replication in control cells, while has no effect in GOT2-knockdown cells. Taken together, these results demonstrate that lncRNA-ACOD1 facilitates viral replication through promoting GOT2 activity.

## Virally Encoded lncRNAs Inhibit Antiviral Responses

The existence of virus-encoded lncRNAs has been identified for years (81, 82). However, only recently, their roles in virus pathobiology and host responses have been explored. The viral lncRNAs are generally transcribed from RNA polymerase II or III, and some of lncRNAs can even be polyadenylated, similar to host mRNA (11, 83). Interestingly, some viral lncRNAs even need unique maturation steps using host cell transcription machineries.

A polyadenylated nuclear RNA (lncRNA PAN) expressed by Kaposi’s sarcoma-associated herpesvirus is localized within the cell nucleus and accumulated largely during lytic infection. Several studies demonstrate that PAN represses host gene transcription through a variety of mechanisms. Interferon regulatory factor 4 (IRF4) is a transcription factor that can bind to and transactivate the IL-4 promoter along with PU.1 (84). However, the expression of PAN interferes with the transcription of IL-4 through preventing PU.1 binding to IL-4 promoter (85). In addition, the results also suggest that PAN decreases the expression of several immune regulators, including IL-18, RNase L, IFN-16, and IFN-γ. This mechanism is closely connected to the extensive binding capacity of PAN and host transcriptional proteins, such as histones H1 and H2A, and mitochondrial and cellular single-stranded binding proteins. Another similar study indicates that PAN suppresses the expression of host antiviral genes by activating the PRC2 (83). Besides broadly inhibiting actions of immunity-related genes, PAN also participates in regulating the virus life cycle. In this context, it has been shown that PAN is able to bind to ubiquitously transcribed tetratricopeptide repeat X chromosome (UTX) and jumonji domain containing 3 (JMJD3) to remove the H3K23me3 from the viral genome, resulting in the change of virus life cycle from latent to lytic infection (86, 87). In addition, PAN interacts with the latency-associated nuclear antigen protein (LANA) to maintain latent infection. Collectively, the viral lncRNA PAN regulates both host and viral gene expression to inhibit antiviral responses and regulate virus life cycle.

Another lncRNA Beta2.7, transcribed from the human cytomegalovirus genome, exists at the early stages of viral infection (88, 89). Beta2.7 and GRIM19 (gene associated with retinoid/IFN-induced mortality-19) are combined together to form a subunit of mitochondrial complex I, which is key for stabilizing the mitochondrial membrane potential, leading to continued production of adenosine triphosphate, which is critical for the completion of the virus life cycle (90–92). Beta2.7 may also protect mitochondrial complex I against stress-induced apoptosis and prevent neuron death.

The 5′-3′ exonuclease Xrn1 functions in mRNA decay as well as degradation of flavivirus genomic RNA (84, 93). Most of the RNAs, even the ones with strong secondary or tertiary structures, cannot resist Xrn1 degradation. Surprisingly, the subgenomic flavivirus RNAs (sfRNAs), generated from viral genome, accumulate to a high level in cells and repress the activation of Xrn1 (94–96). A further study demonstrates that the lncRNA sfRNAs are transcribed at the 3′-terminus of flavivirus genome. Based on the special stem-loop structure, the lncRNA sfRNAs bind to the Xrn1 and inhibit its cascade function. Moreover, Xrn1 can also be used to form new 5′-terminus of transcripts to improve viral gene expression *via* the generation of the lncRNA sfRNAs (95). The lncRNAs from hepaciviruses (e.g., HCV) and pestiviruses (e.g., bovine viral diarrhea virus) are shorter than those from arthropod-borne flaviviruses, which implies that they may play unique roles in the virus life cycle. The transcription and function of the lncRNA sfRNAs indicate that flaviviruses repress host immune system with virus-encoded lncRNAs (Table 1).

**Table 1:** Characteristics of lncRNAs involved in host–virus interactions.

Functions of lncRNAs	Names	Mechanisms	Sources	References
Antiviral responses	NeST	NeST interacts with WDR5 to alter histone 3 methylation at the IFN-γ locus to induce the IFN-γ expression	Host	(72)
	
	NRAV	NRAV inhibits the initial transcription of IFITM3 and MxA by regulating the histone modifications of these ISG genes	Host	(62)
	
	lncRNA#32	lncRNA#32 significantly increases the expression of IRF7, CCL5, CXCL11, OASL, RSAD2, and IP-10 through its interaction with ATF2 and hnRNPU	Host	(68)
	
	BISPR	BISPR induces the transcription of BST2 gene in trans by counteracting the repressive action of PRC2	Host	(56)
	
	Cox2	Cox2, induced by TLR, can interact with hnRNP-A/B and hnRNP-A2/B1 to mediate the immune responses in both positive and negative regulatory signaling pathways	Host	(41)
	
	THRIL	THRIL binds to hnRNPL and TNF promoter/enhancer region to induce TNF-α expression and is downregulated by TNF activation through a negative feedback mechanism	Host	(38)
	
	NEAT1	NEAT1 activates the transcription of IL-8, RIG-I, and DDX60 through removal of the transcriptional inhibitory effects of SFPQ from promoter region by relocating SFPQ to paraspeckles	Host	(34, 44, 46)
	
	CMPK2	lncRNA CMPK2, as a negative regulatory factor in ISGs response, is involved in the regulation of ISGs transcription by forming RNA-protein complexes with chromatin remodeling or transcription factors	Host	(25)
	
Virus infections	NRON	The HIV-1 Nef and Vpu proteins reduce or increase the expression of NRON at different times postinfection to regulate the virus life cycle, resulting in persistent infection	Host	(77)
	
	NeST	Overexpression of NeST has been shown to increase the persistence of Theiler’s virus and reduce the host resistance	Host	(75)
	
	lncRNA-ACOD1	lncRNA-ACOD1 is induced during viral infection and facilitates viral replication through promoting the catalytic activity of GOT2	Host	(80)
	
	PAN	PAN is a key regulator in controlling gene expression by multiple mechanisms. Many immunity-related genes, such as IL-4, IFN-γ, IL-18, and IFN-α, are regulated by lncRNA PAN. In addition, PAN participates in regulating the virus life cycle through removing the suppressive H3K23me3 from the viral genome and interacting with LANA to maintain latent infection	Virus	(83–87)
	
	Beta2.7	Beta2.7 and GRIM19 are combined together to form a subunit of mitochondrial complex I, leading to continued production of adenosine triphosphate	Virus	(92)
	
	sfRNAs	Based on the special secondary structure, sfRNAs bind to Xrn1 and inhibit its degradation of flaviviral genomic RNA	Virus	(94, 95)

*lncRNAs, long non-coding RNAs; WDR5, WD repeat-containing protein 5; IFN-γ, interferon-γ; NRAV, negative regulator of antiviral response; ISG, interferon-stimulated gene; IRF7, interferon regulatory factor 7; BISPR, BST2 IFN-stimulated positive regulator; hnRNPU, heterogeneous nuclear ribonucleoprotein U; ATF2, activating transcription factor 2; PRC2, polycomb repression complex 2; TLR, toll-like receptor; hnRNP, heterogeneous nuclear ribonucleoprotein; THRIL, TNF-α and hnRNPL-related immunoregulatory lncRNA; NEAT1, nuclear enriched abundant transcript 1; HIV, human immunodeficiency virus; GOT2, glutamic-oxaloacetic transaminase 2; sfRNAs, subgenomic flavivirus RNAs*.

## Concluding Remarks and Prospects

Formerly, lncRNAs were considered as non-functional gene transcripts in cells and the studies on host–virus interactions were mainly focus on the genomic DNA and proteins of hosts or viruses. However, in the past few years, powerful evidence supports that some lncRNAs from hosts or viruses are actively involved in host–virus interactions. On one hand, host-encoded lncRNAs are supposed to exert antiviral functions *via* different immune response processes, including innate and adaptive immune responses and ISG expression through completely different mechanisms. On the other hand, viruses seem to hijack host lncRNAs or to exploit viral lncRNAs for inhibition of antiviral responses and virus persistence. Thus, besides DNA and proteins, lncRNAs are a new kind of actors in host immune defense and virus survival.

Here, we raise a question: how to identify functional lncRNAs? To obtain the potential lncRNAs, conventionally researchers analyze the transcriptome and screen the differential expression of mRNAs and lncRNAs induced by viral infections. However, a leading challenge is how to separate lncRNAs from mRNAs in large-scale transcriptome data, since hundreds or even thousands of differentially expressed lncRNAs will be obtained using RNA-seq data, making it laborious to identify functional lncRNAs. Indeed, unlike mRNAs, the sequences of lncRNAs usually display poor evolutionary conservation among different species, thus it is difficult to use conventional bioinformatic tools to predict their functions. In addition, the sequences of lncRNAs are yet to be determined in most species. In spite of these limitations, many lncRNAs from viruses or hosts have been disclosed in recent years. We propose to establish bioinformatics pipelines to genetically annotate lncRNAs by incorporating our current understandings on the functions of lncRNAs in the future.

Since lncRNAs are associated with DNA, mRNA or proteins, it is worth thinking about the possible existence of potential links between lncRNAs and miRNAs. This speculation is supported by some studies that lncRNAs can act as efficient miRNA “sponges” to reduce miRNA levels or through binding to primary miRNAs to repress miRNA maturation (97, 98). However, the discovery about the functions of sncRNAs is scarcely reported in viral infections or host–virus interactions. Up to now, the interactions between miRNA and lncRNAs are a freshly new frontier research area.

Currently, relatively complete lncRNA databases have been established only for human and model animal species (mouse and rat). However, based on the current findings, we believe that lncRNA databases for broader species will facilitate the study on natures and dynamics of lncRNAs-mediated antiviral responses and regulation of the virus life cycle.

In conclusion, growing evidence suggests that additional hosts- or viral-origin lncRNAs remain undiscovered, and systematic and novel probing approaches are required to characterize functional lncRNAs and identify clinically relevant lncRNAs with broader antiviral characteristics.

## Author Contributions

X-YM is the major contributor of the review. YL, MNA, YS, YG, HZ and MM participate in the modification of the article. H-JQ conceived and revised the paper.

## Conflict of Interest Statement

The authors declare that the research was conducted in the absence of any commercial or financial relationships that could be construed as a potential conflict of interest.
